# Changes in Perioperative Antimicrobial and Anti-Inflammatory Drugs Regimens for Colic Surgery in Horses: A Single Center Report

**DOI:** 10.3390/vetsci9100546

**Published:** 2022-10-04

**Authors:** Marco Gandini, Anna Cerullo, Paolo Franci, Gessica Giusto

**Affiliations:** Department of Veterinary Sciences, University of Turin, 10095 Grugliasco, Italy

**Keywords:** colic surgery, horse, perioperative antimicrobial protocol, perioperative anti-inflammatory protocol, postoperative complications

## Abstract

**Simple Summary:**

The administration of postoperative anti-inflammatory and antimicrobial drugs after colic surgery is based on an empirical approach, and for this reason, in recent years, it has been questioned. Recent guidelines recommend that antimicrobials should be administered for the shortest effective period possible. The use of non-steroidal anti-inflammatory drugs is also discussed given the side effects especially on the gastrointestinal tract. Consequently, the antimicrobial and anti-inflammatory drugs administration in horses has changed in our practice over the years to modulate therapies according to the postoperative complications that eventually arise. The description of these changes and the reasons behind them can help define an appropriate stewardship. Over the years, the administration of postoperative antibiotics has been limited, and treatments have been started only in case of complications that justified their use. As for anti-inflammatories, there was a variation of dosages of flunixin meglumine and the addition of new types of anti-inflammatories, both non-steroidal anti-inflammatory drugs and corticosteroids. These changes in prophylaxis protocols were not associated with an increase in postoperative complications.

**Abstract:**

Reducing postoperative incisional infection is the main reason to administer postoperative antimicrobials (AMD) after emergency laparotomy in horses, while reducing inflammation and providing analgesia are the reasons to administer anti-inflammatory drugs (AID). The basis for postoperative AMD and AID administration is empirical and only recently has been questioned. Empirical approaches can be changed, and these changes, along with the description of their outcomes, can help produce appropriate stewardship. The aim of this study is to report the changes in AMD and AID regimens in horses undergoing emergency laparotomy at a referral teaching hospital between 2017 and 2021. Signalment, pathology, surgery, prophylactic AMD and AID administration were obtained from the medical records. Difference in AMD and AID regimens throughout the study period were also reported. In 234 postoperative records considered, ninety-two horses received prophylactic AMD, while 142 received pre-operative antimicrobials only. There was a progressive change in regimens throughout the years, increasing the number of AID molecules used. AMD and AID administration in horses has changed in our practice over the years to modulate therapies according to the postoperative complications that eventually arise. In this study, horses not receiving postoperative routine AMD treatment did not show an increased incidence of complications.

## 1. Introduction

Emergency laparotomy in horses can be classified mostly as clean-contaminated surgery and in some cases even a clean surgery [1]. Nevertheless, bacterial infections, particularly surgical site infection (SSI) and thrombophlebitis, are part of the bulk of postoperative complications after colic surgery and increase hospitalization length, management costs, morbidity and mortality [2]. Reducing postoperative incisional infection is the main reason to administer postoperative antimicrobials [3], while pyrexia is reported as an outcome in the evaluation of the efficacy of antimicrobial drugs (AMDs) administration [4]. The administration of antibiotics pre- and postoperatively is routine in colic surgery, and along with anti-inflammatories and the aseptic preparation technique of the operating field, it has been described to reduce the risk of surgical site infections, peritonitis and postoperative adhesions [1]. Nevertheless, the basis for both postoperative anti-inflammatory and antimicrobial administration is empirical and only recently has been questioned. Guidelines for the judicious use of antimicrobial therapy recommend that antimicrobials should be administered for the shortest effective period possible in order to prevent the development of resistant pathogens and therefore should be avoided in cases where it is not strictly necessary [5,6]. Likewise, the extensive use of NSAIDs has also been questioned given the detrimental side effects on the urinary and gastrointestinal tract [7] and on wound heling. A prospective study comparing the administration of antimicrobials either for three or five postoperative days found no difference in the occurrence of incisional infections after colic surgery [8], and a retrospective study found no difference in the incisional infection rates for horses undergoing colic surgery and administered perioperative antimicrobial drugs for less than 36 h compared to horses administered antimicrobial drugs for more than 36 h. Although not significant, there was a higher prevalence of infection in horses receiving more than 36 h of antimicrobials administration [3]. Recent studies confirmed that the reduction in antibiotics administration after colic surgery is not associated with an increase in infectious complications [9] and that preoperative administration only is comparable to a 5-day postoperative course [10]. Furthermore, prolonged prophylactic antibiotic therapy may increase the risk of antibiotic-induced colitis [11] and thrombophlebitis, which is why some authors have emphasized the need for responsible antimicrobial use, whereas [12] some authors even questioned if it was appropriate to administer postoperative antibiotics at all [2]. The postoperative administration of non-steroidal anti-inflammatory drugs (NSAIDs) may also have side effects that can be detrimental in the postoperative period, and therefore, research of other regimens has been carried out, although the most used protocol is based on flunixin meglumine [7]. Empirical approaches to antimicrobial regimens can be changed, and these changes, along with the description of their outcome, can help produce appropriate antimicrobial stewardship [13,14]. Changes in NSAIDs protocols may lead to a goal-directed therapy aimed at preserving positive effects of postoperative inflammation whilst providing analgesia and reducing their side effects. The aim of this study is to report the change in the antimicrobial and anti-inflammatory regimen in horses undergoing emergency laparotomy at a referral teaching hospital.

## 2. Materials and Methods

This is a retrospective, single-center study describing the use of perioperative AMDs and AIDs in horses that have undergone colic surgery. Medical records of horses admitted to the Veterinary Teaching Hospital of Turin between January 2017 and April 2021 that underwent colic surgery and recovered from anesthesia were retrieved. Horses of all breeds, sexes or ages were included, except for foals younger than 1 year, which were excluded. Horses submitted to pre- or intra-operative euthanasia or death, donkeys and mules were excluded. The medical records of horses were analyzed, and data on signalment (sex, age, weight), pathology (large/small intestine involved, strangulating/non strangulating lesion), type of surgery (clean or clean–contaminated, depending on whether an enterotomy and/or anastomosis was performed), perioperative antimicrobial and anti-inflammatory regimen, time from antimicrobial administration to first incision and surgery duration were retrieved. The occurrence of postoperative pyrexia, which is a clinical sign directly associated with numerous complications (e.g., infection, inflammation, piroplasmosis), postoperative day (POD) on which the first episode of pyrexia occurred, adjunctive AMDs and AIDs administration, the presence of incisional infection or phlebitis, and the results of piroplasmosis testing, given the frequent finding of this infection in the Italian territory, were also recorded. An incisional complication was defined as any incisional drainage present from 12 h or longer after surgery and thrombophlebitis as a warm, painful swelling and cord-like jugular adjacent to the entry point of the venous catheter [15].

### Statistical Analysis

Data were analyzed with Graphpad Prism 9.0. Data were reported as percentage of cases, and a chi-square test or Fisher exact test were used for categorical comparisons (signalment, short-term follow up, pathology, type of surgery, AMD extra prophylaxis protocol and reason, intraoperative dexamethasone, AIDs extra protocol and reason, pyrexia) with significance set for *p* < 0.05.

## 3. Results

### 3.1. Population

Records from horses referred for surgical colic between January 2017 and April 2021 were retrieved. A total of 234 horses were included. Table 1 reports the population and surgical data.

### 3.2. Surgery

All horses were operated on by the same surgeons (MG and GG). Whenever possible, depending on the pain demonstrated by the horse, as soon as consent for surgery was obtained, the abdomen was clipped and the skin washed before entering the induction box. Once anesthesia was induced, the patient was moved from the induction/recovery box to the operating theater, positioned on the operating table, and a 15 min sterile surgical scrub, with soapy chlorhexidine digluconate and alcoholic chlorhexidine, was performed. The operating field was prepared by applying a surgical drape with an adhesive film, and a linea alba incision was performed in all horses. In 25 cases, also a scrotal approach was performed to resolve inguinal hernias. During surgery, the intestine was kept moist with sterile isotonic solution. The abdominal wall was closed in two layers, with a simple continuous pattern of USP 2 Polidioxanone (PDS) loop with a taper needle on the linea alba [16] and horizontal mattress suture of USP 1 Nylon on skin covered with an abdominal stent soaked in povidone–iodine that was maintained in place for 3–5 days; a long-stay sterile jugular catheter was inserted as soon as the horses were moved out of the recovery box. Laparotomy wounds and a jugular catheter insertion point were visually monitored daily after surgery. Ultrasound evaluations were performed only in case of suspected herniation on laparotomy wounds or suspected vascular thrombosis at the jugular level.

### 3.3. Pre and Intra-Operative AMDs and AIDs Administration

All horses received a preoperative administration of flunixin meglumine 1.1 mg/kg IV (Meglufen, IZO Srl, Brescia, Italy) if not already given at admission or by the referring veterinarian in the last 8 h, gentamicin sulfate 6.6 mg/kg IV (Aagent, Fatro S.p.A., Ozzano dell’Emilia, Bologna, Italy) and benzylpenicillin 12,000 UI/kg IM (Depocillina, Msd Animal Health Srl, Sagrate, Milano, Italy) as per label just before induction of anesthesia. The median time between AMDs administration and start of surgery (skin incision) was reported in 193 out of 234 horses as 17 min (range 15–25 min). In two cases that went recumbent during transportation to the hospital and were therefore anesthetized before unloading, AMDs were administered when on the operating table. In these cases, gentamicin sulfate only was administered to avoid the risk of blood pressure reduction from penicillin administration during general anesthesia [17]; because of this, AMDs were also continued in the postoperative period. Intraoperative AMDs redosing was not performed due to the risk of having a lowering of blood pressure, which would make the patient’s anesthetic management more complex [17]. The linea alba was lavaged with Lactated ringer’s solution before completing abdominal closure, but no other treatment was performed. In cases where intestinal edema was considered an issue, a single dose of dexamethasone (1.1 mg/kg IV) was given intraoperatively.

### 3.4. Postoperative AMDs during Hospitalization

**Figure 1:**
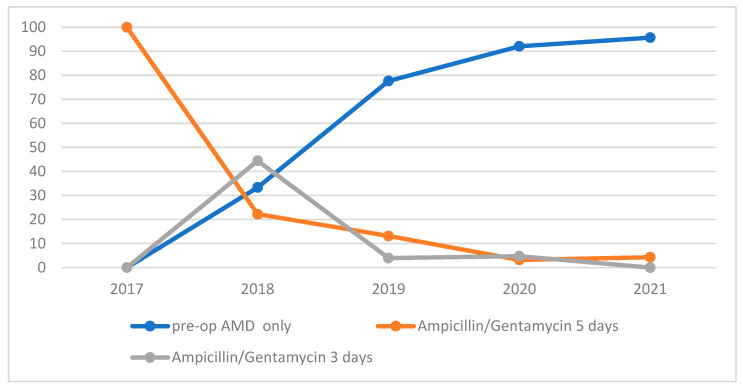
Use of antimicrobial drugs during the years in our referring center.

Ninety-two out of 234 horses received postoperative antimicrobial prophylaxis, of which 70/92 received it for five postoperative days and 22/92 received it for three postoperative days. The remaining horses received only preoperative prophylaxis. Eighty-one out of 234 horses received extra-prophylactic antimicrobials; of these, thirty in the group of horses had already undergone postoperative antimicrobial prophylaxis and fifty-one horses received no AMDs for prophylaxis. Antimicrobials in addition to those used routinely for prophylaxis were administered for piroplasmosis (suspected or confirmed) in 61/81 horses, for SSI in 11/81 horses, for laminitis in 7/81 horses, for peritonitis in 3/81 horses, for phlebitis in 2/81 horses, and for other reasons (e.g., abortion, pulmonary diseases, duodenitis proximal jejunitis) in 8/81 cases. From 2017 to 2021, there was a progressive change in antibiotic administration protocols, with a notable reduction in the administration of postoperative prophylactic AMDs (Figure 1). The administration of antibiotics in the postoperative period was carried out not for prophylactic purposes but mostly to treat specific problems if necessary (Table 2).

### 3.5. Postoperative AIDs Drugs Administration during Hospitalization

**Figure 2:**
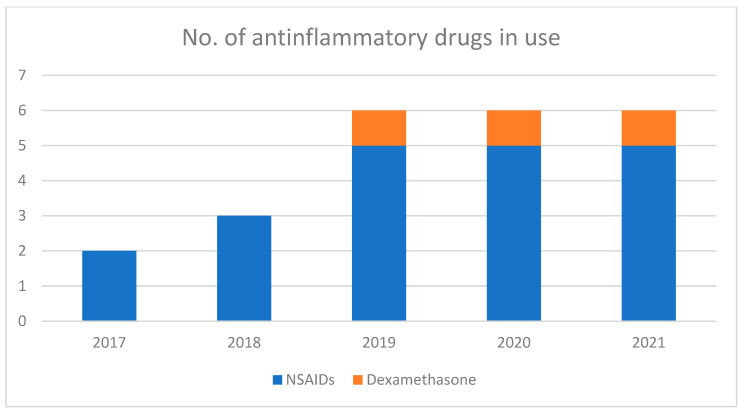
Number of anti-inflammatory drugs or dosages administered postoperatively from postoperative day three over the standard, initial postoperative regimens (flunixin meglumine 0.5 mg/kg IV BID, flunixin meglumine 1.1 mg/kg IV BID, ketoprofen 2.2 mg/kg SID, phenylbutazone 2.2 mg/kg SID, dexamethasone 1.1 g/kg single dose intra-operative).

**Figure 3:**
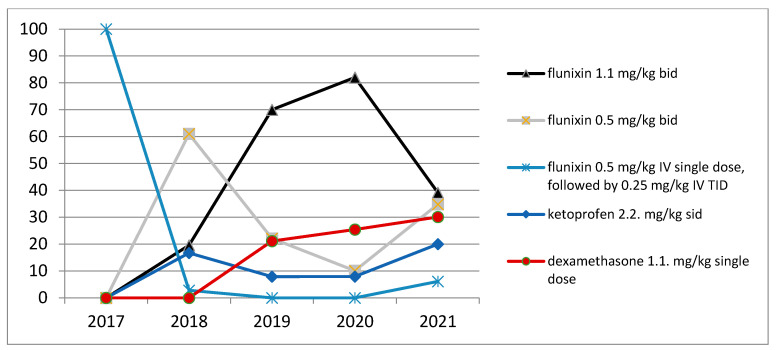
Use of anti-inflammatory drugs during the years in our referring center.

All horses received anti-inflammatory therapy for 3 days: of these, 45 horses received low doses of flunixin meglumine (0.5 mg/kg IV single dose postoperatively, followed by 0.25 mg/kg IV TID), 44 horses received flunixin meglumine at 0.5 mg/kg IV BID, 122 horses received flunixin meglumine at 1.1 mg/kg IV BID and 23 horses received ketoprofen at 2.2 mg/kg SID (Table 3). From 2017 to 2021, there was a progressive change in the administration protocols of anti-inflammatories, with a change in the dosages of flunixin meglumine and the addition of new types of anti-inflammatories, both NSAIDs and corticosteroids (Figure 2 and Figure 3). Over the years, the administration of various types of drugs has been performed based on the problems suspected or encountered in the postoperative period.

### 3.6. Short-Term Follow Up

#### Complications

Despite the changes in the AMDs and AIDs protocols administered over the years, there were no significant differences between the groups in relation to the onset of fever (Table 4), infection and other postoperative complications (Table 2 and Table 4). Twenty-four horses died or were euthanized postoperatively (seven for postoperative colic, six for adhesions, four for postoperative ileus, two for endotoxemia, three for peritonitis and one for hemorrhage).

## 4. Discussion

The problem of antibiotic resistance is growing, and it is essential to use antibiotics prudently, maximizing the therapeutic benefits and minimizing any negative impact on public health [5,12,18]. Antimicrobial stewardship in horses is receiving increasing attention because of the impact of antimicrobial resistance [5,12,18]. To optimize the administration of AMDs and AIDs, it is essential to describe their use in horses undergoing colic surgery and to evaluate their effects in the postoperative period. Colic surgery can present various postoperative complications, which can be partly prevented by the administration of AMDs and AIDs. However, the administration of perioperative antibiotics has, over the years, led to a progressive greater resistance, with an increase in the Minimum Inhibitory Concentration (MIC) for different bacteria [18] and increase in the risk of development of multiresistant bacteria [5]. It is therefore essential to investigate whether there is an actual clinical need to administer antibiotics to colic cases. De-escalation from an empirical antimicrobial regimen administered for purposes of disease treatment, ideally guided by knowledge of the pathogen and an antimicrobial susceptibility report, is a major tenet of judicious antimicrobial use.

In this study, we report the changes in AMDs and AIDs administration in horses submitted to colic surgery during a five-year period in a single referring center. Throughout the years, we started using preoperative AMDs more and were more selective about using less postoperative AMDs in a prophylactic manner There was also a progressive change in AIDs regimens, with an increase in dosage of flunixin meglumine and an increase in the number of AIDs used in the postoperative period. Intraoperative dexamethasone was also introduced in selected cases from 2019, where intestinal edema was considered an issue. These changes were made to maintain the benefits of administering AMDs and AIDs, when needed, while limiting their side effects. Despite these changes, there was no difference in incidence of complications between groups.

AMDs alone are not effective in reducing SSI. Good prophylaxis is not strictly linked to the administration of drugs but must also be accompanied by other measures. It is important to choose the right administration time and to adhere to the rules of good surgical practice to limit contamination of the operating field and therefore the risk of infection. In our study, preoperative antimicrobial prophylaxis was performed within one hour of the skin incision, and intraoperative AMDs redosing was not performed due to the risk of having a lowering of blood pressure, which would make the patient’s anesthetic management more complex [17]. In our hospital, clipping and washing of the abdomen are performed, whenever possible, before taking the horse in the induction box, reducing the time between preoperative antibiotics administration and surgical incision. Meticulous isolation of anastomotic and enterotomy sites from the surgical field with combined absorbent-waterproof barriers concur to reduce the contamination of the procedure. Incisional lavage with sterile saline prior to skin closure has been shown to have a protective effect against SSI development in exploratory celiotomies [19]. While topical antimicrobial use is supported in small studies, a meta-analysis of topical antimicrobial therapy for the prevention of SSI in humans found insufficient evidence for their use in clean and contaminated procedures [20]; for this reason, this practice has not been applied in recent years in our center. In our opinion, all these measures allowed reducing the use of AMDs while maintaining the same rate of complications.

The surgical site infection rate in our caseload was 25.2% in accordance with previous works reporting data from horses undergoing laparotomy and treated with postoperative antibiotics [8,21,22,23,24], and we found no differences between subjects who received antimicrobial prophylaxis and subjects who did not.

In our study, twenty-one horses out of 59 developed SSI without fever. We can therefore assume that fever in subjects with SSI is not necessarily related to this infection, because horses can also have concomitant problems that cause fever, such as piroplasmosis, thrombophlebitis or postoperative inflammation. In our practice, we decided to reduce antimicrobial regimens after noticing that with a five-day postoperative antimicrobial course, many horses still had fever in absence of other signs of infection. This finding was considered due to pyrexia mostly due to surgical inflammation or piroplasmosis that is endemic in our area. Piroplasmosis should be suspected in horses undergoing colic surgery and that present fever of unknown origin in the postoperative period [25]. Whether horses undergoing colic surgery, affected by piroplasmosis, tend to develop more complications or higher mortality has never been investigated and could be the scope of future studies. In cases where other complications arose (piroplasmosis, colitis, laminitis, abortion, refractory SSI with positive culture), we used extra-prophylactic AMDs. In cases of laminitis, oxytetracyclines were administered for their effect in inhibiting metalloproteinases [26]. In our study, two horses in which postoperative prophylaxis was not immediately started after surgery died of postoperative peritonitis; however, we believe that the administration on postoperative antimicrobials would not have avoided this complication, since its onset is most likely a consequence of continued intestinal necrosis or of anastomosis leakage. The percentage of thrombophlebitis in our study was lower than that reported in the literature (5.5% compared to reported 7.5 to 10%) [27], even though an intravenous catheter was maintained in place for 5–7 days postoperatively [8]. This could be due to less manipulation of the intravenous catheter when not administering postoperative antibiotics.

Even the administration of AIDs could have side effects, especially on the gastrointestinal system. Our work highlights the need to investigate the differences given using different anti-inflammatory protocols and to apply AIDs protocols maximizing the benefits while limiting the side effects. If necessary, NSAIDs might be replaced by other anti-inflammatory drugs, analgesics and antipyretics to reduce the side effects on the gastrointestinal system [28]. In human medicine [29,30], NSAIDs have been replaced by opioids, which have less effects on mucosal heling and ulceration. In horses, studies on the effects of more COX-2-selective NSAIDs such as meloxicam and firocoxib have been performed but, to date, there is not enough evidence of them being more beneficial than flunixin meglumine [31,32].

In our study, the rate of postoperative fever is 59%. As suggested by Freeman and colleagues (2012), slight to mild fever (38–39.4 °C) in the early postoperative period is not necessarily associated with an impending bacterial infection in colic cases but rather is due to inflammation [4]. The pyrexia in the first 48 postoperative hours may therefore not be influenced by antibiotics therapy but rather by the administration and dosage of antipyretic and anti-inflammatory drugs. Hence, this can explain differences in complication rates, especially when considering pyrexia as an outcome of antimicrobial therapy. The most used protocol in postoperative colic surgery involves a three-day course of flunixin meglumine BID, which could mask the onset of fever because it attenuates the inflammatory component typical of the immediate postoperative period [4]. In a horse with pyrexia, flunixin meglumine may not be the most suitable AID to use because of its scarce antipyretic effect and its relevant side effects on the gastrointestinal system. Furthermore, the recommended dose of flunixin meglumine for the postoperative management of colic horses (1.1 mg/kg BID IV) is double the label dose [32]. These considerations guided our changes in AID regimens, increasing the number of anti-inflammatory molecules used and setting different dosages according to the clinical cases. Recommendations for the use of NSAIDs in people are to take to lowest effective dose for the shortest period possible to control symptoms. Therefore, even for horses in critical conditions, the application of this practice can only be advantageous [7].

This study has some limitations. Firstly, the selection of animals was not blinded, as was the surgeon to choose if preoperative administration was sufficient or was followed by postoperative prophylaxis or extra-prophylactic AMDs administration. Furthermore, we did not analyze the impact of the different protocols on the development of adhesions, as the onset of adhesions can only be detected with a second laparotomy or post-mortem necropsy. In addition, we did not score the postoperative pain with different AIDs protocols. While the timing of preoperative administration of AMD is important, intraoperative redosing is recommended to avoid underdosing. None of our cases received an intraoperative dose of AMD due to the anesthetists’ fear of a sudden drop in blood pressure [17].

The reason for administering antibiotics and anti-inflammatories is crucial to understand the optimal protocol to apply considering that both AMDs and AIDs could have side effects. Changing AMDs and AIDs regimens is not easy, since standard procedures have been followed for years in horses undergoing emergency laparotomy, and there is a lack of literature on this matter. The difficulty in changing, as Muntwyler and colleagues [14] also report, is related to the fear that a deviation from common administration protocols could cause an increase in postoperative complications; on the other hand, it is easier to accept the side effects of using AMD and AID rather than the risk of complications that are thought to occur in the event of non-treatment [14].

## 5. Conclusions

In conclusion, surveillance of the use of AMDs and AIDs is essential for their management and can provide important information for evaluating current practices, identifying any problems in applying a particular protocol and then modifying them based on their effectiveness on individual clinical cases. The basis for both postoperative AMDs and AIDs administration in colic surgery is empirical. Nevertheless, this empirical approaches to administration regimens can be changed, and these changes, along with the description of their outcome, can help produce appropriate AMDs and AIDs stewardship. The administration of postoperative AMDs does not appear to be indicated in all horses, just as the administration of AIDs can be modulated to obtain the best anti-inflammatory effect and reduce side effects without an increased incidence of complications. However, randomized case-control studies are needed to define the best AMDs and AIDs postoperative regimen for colic surgeries.

## Figures and Tables

**Table 1:** Population and surgical data of horses referred for surgical colic between January 2017 and April 2021.

		AMD		No AMD		Total			
		*n*	%	*n*	%	*n*	%	Test	*p*
Total cases		92		142		234			
	Postoperative euthanasia	14	15.2	17	12	31	13.3	Chi-square	0.51
	Discharged	78	84.8	125	88	203	86.7		
Female		35	38	54	38	89	38	Chi-square	0.99
Gelding		41	44.6	67	47.2	108	46.2		
Stallion		16	17.4	21	14.8	37	15.8		
Pathology	Small intestine—Strangulating lesions	23	25	33	23.2	56	24	Chi-square	1
	Small intestine—Non-Strangulating lesions	11	12	25	17.6	36	15.4		
	Large intestine—Strangulating lesions	12	13	16	11.3	28	12	Chi-square	0.06
	Large intestine—Non-Strangulating lesions	46	50	68	47.9	114	48.6		
Enterotomy/anastomosis		80	87	131	92.3	211	90.2	Chi-square	0.18

**Table 2:** Data of antimicrobial administration in the postoperative period and reason behind this approach. (SSI: surgical site infection; DPJ: duodenitis–proximal jejunitis).

		AMD		NO AMD		Total			
		*n*	%	*n*	%	*n*	%	Test	*p*
Number of horses per group		92		142		234			
AMD prophylaxis	5-day course	70		0		70			
	3-day course	22		0		22			
AMD extra prophylaxis protocol		30/92	32.6	51/142	35.9	81/234	34.6	Chi-square	0.603
Reason	SSI	6/30	20	5/51	9.8	11/81	13.6	Chi-square	0.195
	Thrombophlebitis	0		2		2		Fisher exact	0.52
	Piroplasmosis	18/30	60	43/51	84.3	61/81	75.3	Chi-square	0.014
	Peritonitis	0		2		2		Fisher exact	0.52
	Laminitis	3		4		7		Fisher exact	0.7
	Others (DPJ, Abortus)	3		5		8		Fisher exact	1

**Table 3:** Data of anti-inflammatory administration in the postoperative period and reason behind this approach (AIDs extra protocol: AIDs in addition to the AIDs administered as part of the protocol).

		AMD		NO AMD		Total			
		*n*	%	*n*	%	*n*	%	Test	*p*
Number of horses per group		92		142		234			
Intraoperative dexamethasone (0.05 mg/kg)		8/92		30/142		38/234		Chi-square	0.01
AIDs extra protocol		35/92	38	74/142	52.1	109/234	46.6	Chi-square	0.03
	Flunixin	11		6		17		Chi-square	0.001
	Phenylbutazone	19		46		65		Chi-square	0.43
	Ketoprofen	5		20		25		Fisher exact	0.22
	Acetylsalicylate	1		2		3		Fisher exact	1
	Multiple drugs	11		0		11		Fisher exact	0.001
Reason	Pyrexia	21/35	60	50/74	67.6	71/109	65.2	Chi-square	0.024
	Colic	8		15		23		Chi-square	0.76
	Laminitis	5		7		12		Fisher exact	0.5
	Other	1		2		3		Fisher exact	1

**Table 4:** Data about the onset of pyrexia in the postoperative period.

		AMD		NO AMD		Total			
		*n*	%	*n*	%	*n*	%	Test	*p*
Number of horses per group		92		142		234			
Pyrexia		40	43.5	63	44.4	103	44	Chi-square	0.893
Duration (hours)	<48	31	77.5	39	61.9	70	68	Chi-square	0.098
°C	<39.5	31	77.5	53	84.1	84	81.6	Chi-square	0.398
Enterotomy/anastomosis + pyrexia		32/80	40	56/131	42.8	88/211	41.8	Chi-square	0.694

## Data Availability

The data presented in this study are available on request from the corresponding author.
